# Association between volatile organic compounds exposure and cardiometabolic function: a population-based study

**DOI:** 10.3389/fpubh.2025.1570752

**Published:** 2025-04-23

**Authors:** Qiuyu Wang, Yongping Cao, Fei Ma, Hengyang Zhang, Yuelin Hu, Wenwen Xiao

**Affiliations:** ^1^Department of Electrocardiology, The Second Affiliated Hospital of Wannan Medical College, Wuhu, Anhui, China; ^2^Eastern Theater Command Centers for Disease Control and Prevention, Nanjing, China

**Keywords:** Cardiometabolic Index, volatile organic compounds, lipid, NHANES, mixture exposure

## Abstract

**Background:**

The Cardiometabolic Index (CMI) is a new metric used to assess an individual’s cardiovascular and metabolic status. Volatile Organic Compounds (VOCs) are common environmental pollutants associated with cardiovascular diseases and lipid metabolism disorders. This study aims to investigate the relationship between VOC exposure and CMI.

**Methods:**

Data from NHANES (2011–2020) were used to assess the impact of VOC exposure on cardiometabolic function. Multivariable linear regression was used to analyze the association between VOCs and the CMI. Restricted cubic spline models were applied to model the nonlinear relationship between VOCs and CMI. BKMR (bayesian kernel machine regression), WQS (weighted quantile sum), and Q-gcomp (quantile g-computation) models were employed to explore the association between VOC mixture exposure and CMI. Subgroup analyses were conducted to investigate the relationship between VOCs and CMI across different subgroups.

**Results:**

Multiple linear regression analysis confirmed the significantly positive associations between the highest quartile concentrations of CEMA, 3HPMA, MHBMA3, and HMPMA compared to the lowest quartile (*β* = 0.43, 95% CI = 0.20, 0.67, P for trend < 0.001; *β* = 0.30, 95% CI = 0.05, 0.55, P for trend = 0.006; *β* = 0.37, 95% CI = 0.14, 0.61, P for trend < 0.001; *β* = 0.28, 95% CI = 0.01, 0.55, P for trend = 0.010). AAMC and SBMA showed a nonlinear relationship with CMI. Results from mixture exposure models indicated that CEMA contributed most significantly to the impact on CMI. BKMR, WQS, and Q-gcomp models showed a positive trend between overall VOC exposure and CMI. Subgroup analysis revealed significant interactions of BMI and diabetes status in the relationship between VOC exposure and CMI, especially among individuals with BMI ≥ 30 and those with diabetes.

**Conclusion:**

This study found significant positive associations between specific VOC exposures and CMI. Additionally, BMI and diabetes status play important roles in moderating the relationship between VOC exposure and CMI. These findings highlight the potential impact of environmental VOCs on cardiovascular and metabolic health which provides new evidence for public health interventions.

## 1 Introduction

Normal cardiometabolic function dynamically regulates the balance between energy supply and demand, ensuring that the heart responds effectively to physical activity, metabolic changes, and environmental stressors (1). The Cardiometabolic Index (CMI) is an emerging composite indicator, providing an accurate assessment of the synergistic effects of lipid metabolism disorders and central obesity (2). Compared to traditional single biomarkers (such as TG and HDL-C) or isolated anthropometric indicators (such as BMI and waist circumference), CMI not only captures dyslipidemia (TG/HDL-C ratio) but also reflects visceral fat distribution (WHtR), providing a more comprehensive assessment of cardiometabolic dysfunction (3–5). The combination of high TG and low HDL-C exacerbates atherosclerosis, while central obesity further amplifies this risk through adipose tissue inflammation (6, 7). Recent studies have demonstrated that CMI outperforms traditional markers (such as LDL-C or waist circumference alone) in predicting metabolic syndrome, insulin resistance, and cardiovascular events (8, 9). This predictive advantage arises from CMI’s ability to quantify the lipid-obesity interaction, a core mechanism underlying cardiometabolic disease. Higher CMI levels are closely associated with a higher risk of developing these chronic diseases, highlighting the importance of regular CMI assessments for early detection and intervention. Monitoring CMI can thus enable healthcare providers to identify individuals at high risk of metabolic disorders and initiate personalized strategies to prevent or manage such conditions.

A variety of factors influence cardiac metabolic function, including genetic predisposition, lifestyle behaviors (such as alcohol consumption and smoking), age, gender, and the presence of chronic conditions like hypertension and diabetes (10–12). In recent years, environmental factors have gained significant attention as contributors to metabolic dysfunction (13, 14). Among these, exposure to chemicals found in everyday environments has emerged as a notable concern. Volatile organic compounds (VOCs) are a class of chemicals that can easily evaporate into the air at room temperature and are commonly found in household products, such as cleaning agents, paints, air fresheners, and cosmetics (15, 16). VOCs have been shown to have potential adverse effects on human health, particularly in relation to fat metabolism and lipid levels (17). Studies suggest that exposure to VOCs can alter metabolic pathways, leading to increased fat accumulation, particularly in the abdominal and visceral regions, which are strongly associated with metabolic disorders (18). VOCs exposure has been linked to an increased body fat percentage and symptoms like abdominal obesity, insulin resistance, and impaired glucose metabolism (19, 20). Additionally, VOC exposure has been shown to elevate concentrations of TG and LDL-C, while simultaneously decreasing HDL-C levels, which is detrimental to cardiovascular health (21). Furthermore, VOCs may interfere with liver lipid metabolism, impairing the synthesis of HDL-C and contributing to metabolic dysfunction (22, 23). These effects suggest that body composition indicators, such as waist circumference and body fat percentage, as well as lipid profiles, could serve as key markers for understanding the impact of VOCs on cardiac metabolic function (21, 22).

However, research on the effects of VOCs on cardiometabolic function is currently limited, and the potential impact of VOCs on cardiometabolic health remains unclear. To address this gap, we utilized data from the National Health and Nutrition Examination Survey (NHANES) collected between 2011 and 2020. This study will provide important epidemiologic evidence to elucidate the effects of environmental VOCs exposure on cardiometabolic health and provide a scientific basis for targeted protection strategies for high-risk populations (obese and diabetic patients), which is important for promoting environmental health risk assessment and public health interventions.

## 2 Method

### 2.1 Study population

The NHANES is a comprehensive, ongoing study conducted by the Centers for Disease Control and Prevention to assess the health, nutrition, environmental exposures, and socio-economic conditions of the U.S. population. NHANES data are collected from a representative sample of individuals across different demographics, and socio-economic status, with the goal of providing valuable insights into public health trends, nutrition, and environmental factors influencing the population’s health. The survey combines interviews, physical examinations, and laboratory tests, which make it a key resource for assessing the health status of the U.S. population and guiding public health policy and clinical decision-making.

**Figure 1:**
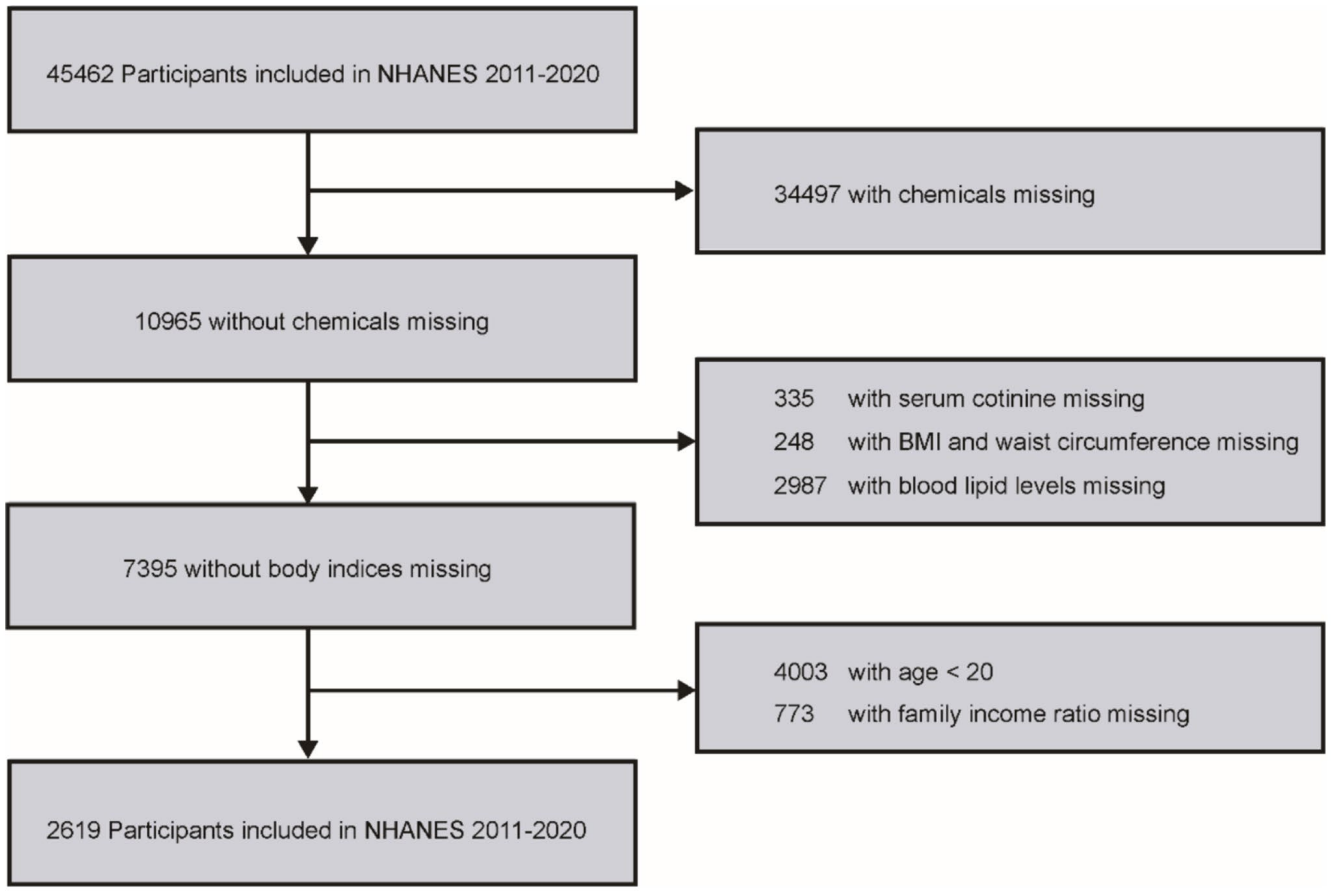
Flow chart for inclusion and exclusion of study participants.

This study used data from a total of 45,462 participants from the NHANES 2011–2020 dataset. Participants were selected based on specific eligibility criteria, and those who did not meet these criteria were excluded. The criteria included: (1) detectable levels of VOCs in urine; (2) age over 20 years; (3) missing or incomplete serum cotinine data; (4) complete family income-to-poverty ratio information; (5) available body mass index (BMI) and waist circumference measurements; and (6) complete data on blood lipid profiles. A detailed flowchart summarizing the inclusion and exclusion process is provided in Figure 1.

### 2.2 Measurement of VOCs

VOCs in urine were measured using ultra-performance liquid chromatography coupled with electrospray tandem mass spectrometry, which is a highly sensitive and accurate technique for detecting and quantifying various volatile organic compounds. In this study, VOCs were assessed as either parent compounds or their metabolites, based on their prevalence and detectability in the sample population. VOCs that were detected in at least 70% of the participants were included in the analysis to ensure robust and reliable results. A list of the VOCs and their abbreviations included in this study is presented in Supplementary Table S1. For VOC levels that were below the limit of detection (LOD), we assigned values using a substitution method where the recorded value was set to the square root of the LOD divided by two to account for missing data.

### 2.3 Outcomes and covariates

The primary outcome in this study is the CMI, a novel metric designed to evaluate visceral fat distribution and associated dysfunction. CMI is a composite measure that provides valuable insights into cardiometabolic health by combining lipid levels (triglycerides and high-density lipoprotein cholesterol) with the waist-to-height ratio, a known indicator of abdominal adiposity. The formula for calculating CMI is as follows (24):

$$\begin{array}{l} CMI=\mathrm{Triglycerides}\left(TG\right)/\mathrm{High}-\mathrm{density}\;\mathrm{lipoprotein}\;\\ \mathrm{cholesterol}\;\left(HDL-C\right)\times \mathrm{WHtR}\;\\ \left(\mathrm{WHtR}=\mathrm{waist}\;\mathrm{circumference}/\mathrm{height}\right) \end{array}$$

In addition to CMI, various covariates were included in the statistical models to adjust for potential confounding factors. These covariates encompassed demographic characteristics and behavioral factors. Clinical factors such as hypertension, diabetes, and BMI, as well as lipid profiles including TG and LDL, were also included. Hypertension and diabetes were assessed based on self-reported diagnoses from a structured questionnaire administered to participants.

### 2.4 Statistical analysis

Descriptive statistics were used to summarize the data. For continuous variables, means and standard deviations were reported, while categorical variables were summarized with counts and percentages. VOC concentrations were log-transformed to normalize their distributions and reduce the skewness often observed in environmental exposure data. To explore the relationships between VOC exposure and CMI, we applied multiple linear regression models. Model 1 was adjusted for basic demographic factors. Model 2 included additional adjustments for lifestyle factors, disease status and clinical conditions, as well as BMI and lipid levels. Effect estimates from these models were presented as *β* values with 95% confidence intervals. To assess the nonlinear relationship between VOCs and CMI, we employed restricted cubic splines (RCS) model with three knots. This flexible approach allowed us to model potential non-linearities in the data and better capture the complex associations between VOC exposure and CMI. The x-axis represented the natural logarithm of VOC concentrations, and the y-axis depicted the estimated effect of CMI. Given the potential for mixed VOC exposures in real-life environments, we utilized several advanced statistical models to assess the combined effects of VOCs on CMI. These models included the Bayesian kernel machine regression (BKMR), weighted quantile sum (WQS) regression, and quantile g-computation (Q-gcomp) models. The BKMR model enabled us to estimate both the overall and individual effects of each VOC exposure, along with the dose–response relationships. The WQS model quantified the relative contribution of each VOC to the overall health effect, while the Q-gcomp model allowed for the estimation of the linear relationship between the combined VOC exposure and CMI. We also conducted subgroup analyses to investigate potential effect modifications by various demographic and health-related factors. These subgroup analyses helped to identify whether the associations between VOCs and CMI varied across different population groups. In order to explain the problem of multiple comparisons, we use Benjamin Hochberg method to adjust the *p* value of multiple comparisons by applying error detection rate (FDR) correction. All statistical analyses were performed using R software (version 4.3.2). A *p*-value of less than 0.05 was considered statistically significant. The “bkmr,” “gWQS” and “qgcomp” R packages were used to conduct mixed exposure analyses and assess the combined effects of VOCs on CMI.

## 3 Results

### 3.1 Basic description of participants

Table 1 displays the baseline features of the study population. A total of 2,619 participants were included in the analysis of the association between VOCs and CMI. Baseline information was described based on quartile groupings of CMI. The average age of participants increased gradually from Q1 to Q4. The proportion of females was higher in the lower quartiles. The percentage of non-Hispanic white participants was the highest in all four groups. The average BMI of participants increased from Q1 to Q4, and the proportion of obese individuals also increased. The proportions of smokers, drinkers, and individuals with a history of hypertension and diabetes increased as the quartile number increased. Serum cotinine, total cholesterol, triglycerides, and LDL levels increased across quartiles, while high-density lipoprotein levels decreased.

**Table 1:** Characteristics of the population based on quartile groupings of CMI.

Characteristics	Q1	Q2	Q3	Q4	*p* value
Age (mean (SD))	45.25 (17.81)	50.36 (17.79)	50.62 (16.60)	51.93 (16.03)	<0.001
Gender (%)					<0.001
Male	266 (40.6)	320 (48.9)	339 (51.8)	407 (62.1)	
Female	389 (59.4)	335 (51.1)	315 (48.2)	248 (37.9)	
Race (%)					<0.001
Mexican American	48 (7.3)	81 (12.4)	75 (11.5)	117 (17.9)	
Other Hispanic	52 (7.9)	54 (8.2)	77 (11.8)	83 (12.7)	
Non-Hispanic White	229 (35.0)	253 (38.6)	238 (36.4)	280 (42.7)	
Non-Hispanic Black	188 (28.7)	168 (25.6)	152 (23.2)	87 (13.3)	
Other Race	138 (21.1)	99 (15.1)	112 (17.1)	88 (13.4)	
Education (%)					<0.001
Less than 9th grade	26 (4.0)	51 (7.8)	50 (7.6)	76 (11.6)	
9–11th grade	56 (8.5)	71 (10.8)	92 (14.1)	97 (14.8)	
High school Graduate/GED or equivalent	126 (19.2)	146 (22.3)	166 (25.4)	165 (25.2)	
Some college or AA degree	219 (33.4)	196 (29.9)	199 (30.4)	194 (29.6)	
College graduate or above	227 (34.7)	191 (29.2)	147 (22.5)	122 (18.6)	
Unknown	1 (0.2)	0 (0.0)	0 (0.0)	1 (0.2)	
Family income ratio (mean (SD))	2.76 (1.68)	2.63 (1.65)	2.41 (1.63)	2.32 (1.54)	<0.001
BMI (mean (SD))	24.77 (4.98)	28.65 (6.35)	30.79 (6.79)	32.81 (7.06)	<0.001
<25	381 (58.2)	193 (29.5)	116 (17.7)	67 (10.2)	
25–29.9	183 (27.9)	248 (37.9)	231 (35.3)	191 (29.2)	
≥30	91 (13.9)	214 (32.7)	307 (46.9)	397 (60.6)	
Smoke (%)					<0.001
Yes	251 (38.3)	277 (42.3)	291 (44.5)	337 (51.5)	
No	404 (61.7)	378 (57.7)	362 (55.4)	318 (48.5)	
Unknown	0 (0.0)	0 (0.0)	1 (0.2)	0 (0.0)	
Drink (%)					0.525
Yes	73 (13.5)	79 (15.0)	84 (15.8)	99 (17.2)	
No	468 (86.3)	446 (84.8)	449 (84.2)	476 (82.8)	
Unknown	1 (0.2)	1 (0.2)	0 (0.0)	0 (0.0)	
Hypertension (%)					<0.001
Yes	145 (22.1)	229 (35.0)	266 (40.7)	291 (44.4)	
No	510 (77.9)	425 (64.9)	387 (59.2)	363 (55.4)	
Unknown	0 (0.0)	1 (0.2)	1 (0.2)	1 (0.2)	
Diabetes (%)					<0.001
Yes	31 (4.7)	67 (10.2)	98 (15.0)	169 (25.8)	
No	606 (92.5)	570 (87.0)	531 (81.2)	467 (71.3)	
Unknown	18 (2.7)	18 (2.8)	25 (3.8)	19 (2.9)	
Cotinine [mean (SD)]	56.23 (126.06)	50.98 (123.20)	64.07 (128.82)	67.47 (166.35)	0.121
Total cholesterol [mean (SD)]	177.79 (37.71)	185.23 (38.99)	191.24 (40.92)	198.61 (41.49)	<0.001
LDL [mean (SD)]	97.28 (30.32)	111.79 (33.63)	119.03 (36.46)	118.73 (36.92)	<0.001
Triglyceride [mean (SD)]	52.05 (15.79)	80.59 (19.16)	112.90 (26.01)	193.48 (65.63)	<0.001
HDL [mean (SD)]	70.11 (17.19)	57.32 (11.35)	49.63 (9.28)	41.18 (8.08)	<0.001

Q, quartile; BMI, body mass index; LDL, low density lipoprotein; HDL, high density lipoprotein.

### 3.2 Distribution of VOC

Supplementary Table S2 presents the distribution of VOCs in urine. Apart from BPMA and CYMA, the detection rates for the remaining VOCs exceed 90%. Among them, the detection rates of DHBMA and HMPMA reached 100% in the study population. The Spearman correlation coefficients between VOCs varied widely, ranging from 0.02 to 0.92 (presented in Supplementary Figure S1).

### 3.3 Associations between VOC and CMI

The association between VOC quartiles and CMI is shown in Table 2. These models were adjusted for demographic characteristics and other covariates. In the fully adjusted Model 2, we found significant positive associations between the highest quartile concentrations of CEMA, 3HPMA, MHBMA3, and HMPMA compared to the lowest quartile (*β* = 0.43, 95% CI = 0.20, 0.67, P for trend < 0.001; *β* = 0.30, 95% CI = 0.05, 0.55, P for trend = 0.006; *β* = 0.37, 95% CI = 0.14, 0.61, P for trend < 0.001; *β* = 0.28, 95% CI = 0.01, 0.55, P for trend = 0.010). No association was observed between other VOCs and CMI estimates.

**Table 2:** Multiple linear regression between individual VOCs and CMI.

	Q1	Q2	Q3	Q4	P for trend
2MHA					
Model 1	Ref	0.08 (−0.03, 0.20)	0.05 (−0.08, 0.18)	−0.10 (−0.37, 0.18)	0.835
Model 2	Ref	0.10 (−0.01, 0.21)	0.06 (−0.08, 0.19)	0.04 (−0.22, 0.31)	0.453
3-4MHA					
Model 1	Ref	0.16 (−0.02, 0.35)	0.11 (−0.08, 0.30)	0.16 (−0.09, 0.42)	0.719
Model 2	Ref	0.13 (−0.04, 0.31)	0.10 (−0.08, 0.29)	0.13 (−0.12, 0.39)	0.610
AAMA					
Model 1	Ref	0.23 (0.03, 0.44)	0.22 (0.01, 0.42)	0.19 (−0.08, 0.46)	0.438
Model 2	Ref	0.20 (0.01, 0.39)	0.24 (0.04, 0.43)	0.19 (−0.07, 0.45)	0.145
AMCC					
Model 1	Ref	0.08 (−0.11, 0.27)	0.16 (−0.03, 0.35)	0.30 (0.04, 0.55)	0.008
Model 2	Ref	0.01 (−0.17, 0.19)	0.08 (−0.10, 0.27)	0.22 (−0.05, 0.48)	0.055
ATCA					
Model 1	Ref	0.07 (−0.07, 0.20)	0.09 (−0.06, 0.23)	0.20 (−0.07, 0.46)	0.142
Model 2	Ref	0.04 (−0.09, 0.17)	0.06 (−0.08, 0.20)	0.16 (−0.09, 0.41)	0.234
SBMA					
Model 1	Ref	0.13 (−0.08, 0.33)	0.16 (−0.05, 0.38)	−0.02 (−0.30, 0.26)	0.785
Model 2	Ref	0.14 (−0.05, 0.34)	0.19 (−0.01, 0.40)	0.00 (−0.27, 0.26)	0.602
BPMA					
Model 1	Ref	0.02 (−0.09, 0.13)	−0.07 (−0.20, 0.07)	0.11 (−0.14, 0.36)	0.870
Model 2	Ref	0.01 (−0.09, 0.11)	−0.04 (−0.16, 0.09)	0.16 (−0.08, 0.40)	0.728
CEMA					
Model 1	Ref	0.28 (0.10, 0.45)	0.42 (0.24, 0.59)	0.52 (0.28, 0.76)	< 0.001
Model 2	Ref	0.22 (0.06, 0.39)	0.38 (0.21, 0.55)	0.43 (0.20, 0.67)	< 0.001^*^
CYMA					
Model 1	Ref	0.04 (−0.11, 0.19)	0.09 (−0.05, 0.23)	0.03 (−0.17, 0.22)	0.322
Model 2	Ref	0.08 (−0.07, 0.23)	0.06 (−0.15, 0.26)	0.02 (−0.24, 0.28)	0.649
DHBMA					
Model 1	Ref	0.12 (−0.07, 0.31)	0.23 (0.04, 0.42)	−0.01 (−0.28, 0.26)	0.119
Model 2	Ref	0.06 (−0.12, 0.24)	0.19 (0.01, 0.37)	−0.07 (−0.32, 0.19)	0.100
2HPMA					
Model 1	Ref	0.01 (−0.12, 0.14)	−0.02 (−0.17, 0.13)	−0.11 (−0.37, 0.15)	0.451
Model 2	Ref	0.00 (−0.12, 0.12)	−0.03 (−0.18, 0.11)	−0.07 (−0.32, 0.18)	0.513
3HPMA					
Model 1	Ref	0.25 (0.08, 0.42)	0.29 (0.11, 0.47)	0.31 (0.07, 0.54)	0.015
Model 2	Ref	0.25 (0.09, 0.41)	0.32 (0.14, 0.49)	0.30 (0.05, 0.55)	0.006^*^
MA					
Model 1	Ref	0.19 (0.00, 0.39)	0.26 (0.07, 0.46)	0.16 (−0.12, 0.43)	0.063
Model 2	Ref	0.12 (−0.07, 0.30)	0.19 (0.01, 0.38)	0.00 (−0.27, 0.27)	0.223
MHBMA3					
Model 1	Ref	0.18 (0.05, 0.31)	0.24 (0.07, 0.40)	0.33 (0.13, 0.54)	< 0.001
Model 2	Ref	0.21 (0.09, 0.34)	0.28 (0.11, 0.46)	0.37 (0.14, 0.61)	< 0.001^*^
PGA					
Model 1	Ref	0.02 (−0.17, 0.21)	0.02 (−0.16, 0.21)	−0.02 (−0.28, 0.24)	0.966
Model 2	Ref	−0.04 (−0.21, 0.14)	−0.01 (−0.19, 0.17)	−0.08 (−0.33, 0.18)	0.947
HMPMA					
Model 1	Ref	0.24 (0.04, 0.45)	0.36 (0.14, 0.57)	0.41 (0.15, 0.67)	0.001
Model 2	Ref	0.18 (−0.01, 0.38)	0.29 (0.08, 0.50)	0.28 (0.01, 0.55)	0.010^*^

Q, quartile; Ref, reference. Model 1 adjusted for age, gender, race, education, family income ratio. Model 2 further adjusted for serum cotinine, alcohol, smoke, hypertension, diabetes, TC and LDL. *means significant results.

We treated VOCs as continuous variables and performed multiple linear regression analysis to explore their relationships. The findings, presented in Supplementary Table S3, revealed significant positive associations between AMCC, CEMA, 3HPMA, MHBMA3, HMPMA, and CMI (*β* = 0.07, 95% CI = 0.02, 0.13, *p* = 0.01; *β* = 0.15, 95% CI = 0.09, 0.20, *p* < 0.01; *β* = 0.08, 95% CI = 0.03, 0.14, *p* < 0.01; *β* = 0.10, 95% CI = 0.05, 0.15, *p* < 0.01; *β* = 0.08, 95% CI = 0.02, 0.14, *p* = 0.01). These results are generally consistent with the findings mentioned above.

### 3.4 Non-linear relationship

**Figure 2:**
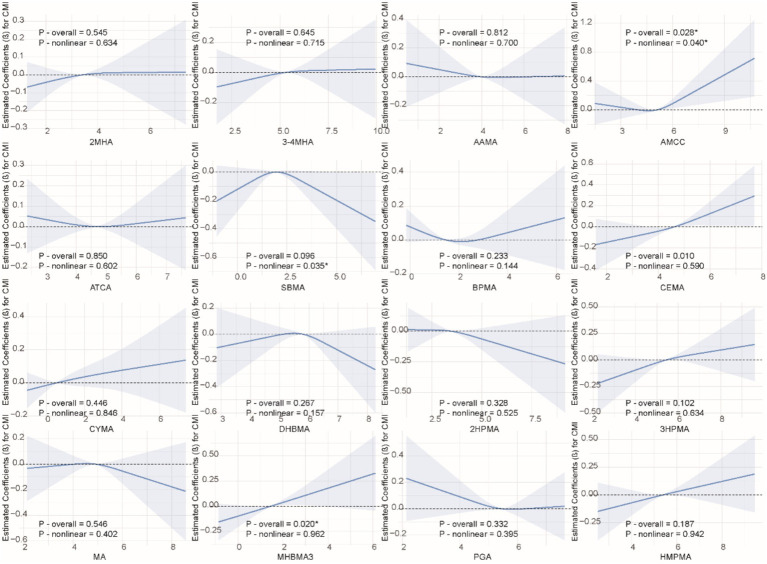
Non-linear relationships between VOCs and CMI. The model was adjusted for age, gender, race, education, family income ratio, serum cotinine, alcohol, smoke, hypertension, diabetes, TC and LDL.

We applied the RCS model to investigate the nonlinear association between VOCs and CMI. As shown in Figure 2, we found significant nonlinear relationships between AAMC, SBMA, and CMI (P-overall = 0.028, P-nonlinearity = 0.040; P-overall = 0.096, P-nonlinearity = 0.035). Additionally, no other significant nonlinear relationships were observed between VOCs and CMI.

### 3.5 Combined effect of VOCs on CMI

**Figure 3:**
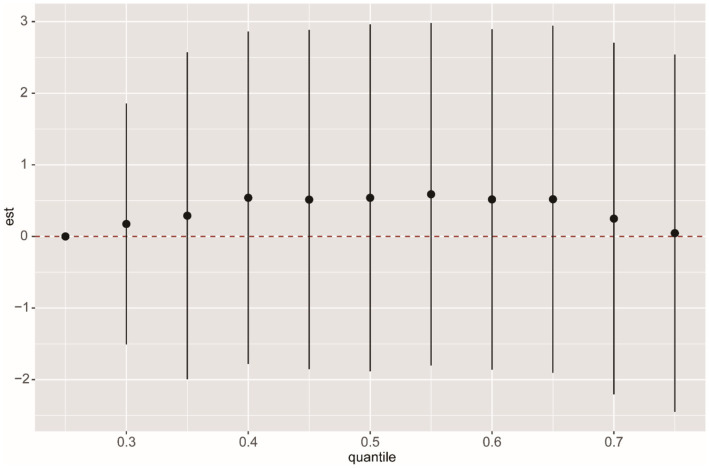
BKMR analysis of the overall effect of VOCs mixed exposure on CMI. The BKMR model uses Gaussian radial basis function kernel, and different percentile chemicals are compared with those in the 25th percentile. “est” can be interpreted as the relationship between VOCs and CMI. The model was adjusted for age, gender, race, education, family income ratio, serum cotinine, alcohol, smoke, hypertension, diabetes, TC and LDL. The sample size in the BKMR model is consistent with the sample size of the linear regression.

**Figure 4:**
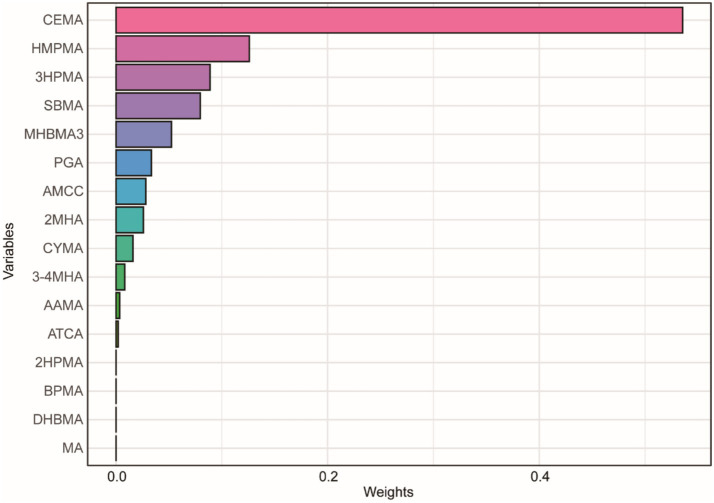
WQS model regression index weights for CMI. The model was adjusted for age, gender, race, education, family income ratio, serum cotinine, alcohol, smoke, hypertension, diabetes, TC and LDL.

We used multiple models to analyze the effects of mixed exposure. The results show that, compared to the 25th percentile, the effect estimates of other percentiles first increased and then decreased, with an overall positive trend, although the differences were not statistically significant (Figure 3). Additional results from the BKMR model are shown in Supplementary Figures S2, S3. In the WQS model, we found that CEMA dominated the mixed effects on CMI (Figure 4). The positive WQS index did not show a significant association with CMI (Supplementary Table S4). The results from the Q-gcomp analysis were generally consistent with those from the BKMR and WQS models (Supplementary Figure S4). CEMA had the highest weight. The linear association between mixed VOCs and CMI showed a positive trend.

### 3.6 Subgroup analysis

We conducted subgroup analyses stratified by variables such as age, gender, race, education level, smoking status, alcohol consumption, hypertension, diabetes, and BMI to examine the effects of VOCs on CMI. The results showed significant interactions between DHBMA, MA, MHBMA, and HMPMA with CMI based on BMI (Table 3). Specifically, MHBMA3 and HMPMA were significantly positively associated with CMI in participants with a BMI ≥ 30 (*β* = 0.18, 95% CI = 0.09, 0.27, *p* < 0.01; *β* = 0.15, 95% CI = 0.05, 0.25, *p* < 0.01). There were also significant interactions between 2MHA, 3-4MHA, AAMA, AMCC, BPMA, CYMA, MA, MHBMA3, PGA, and HMPMA with CMI based on diabetes status. Notably, 2MHA, MA, MHBMA3, and HMPMA were significantly positively associated with CMI in participants with diabetes (*β* = 0.14, 95% CI = 0.00, 0.27, *p* = 0.04; *β* = 0.19, 95% CI = 0.00, 0.38, *p* = 0.05; *β* = 0.23, 95% CI = 0.06, 0.39, *p* = 0.01; *β* = 0.24, 95% CI = 0.07, 0.41, *p* = 0.01). The results of subgroup analyses for other variables are presented in Supplementary Table S5.

**Table 3:** Subgroup analysis for associations between VOC and CMI.

Variables	BMI					Diabetes				
	<30		≥30			Yes		No		
	Estimate (95% CI)	*p* value	Estimate (95% CI)	*p* value	P-int	Estimate (95% CI)	*p* value	Estimate (95% CI)	*p* value	P-int
2MHA	0.01 (−0.04, 0.05)	0.81	0.05 (−0.02, 0.12)	0.19	0.46	0.14 (0.00, 0.27)	0.04^*^	0.00 (−0.04, 0.04)	0.91	<0.01^*^
3-4MHA	0.00 (−0.04, 0.04)	0.90	0.05 (−0.03, 0.12)	0.22	0.32	0.13 (−0.01, 0.28)	0.07	0.00 (−0.04, 0.04)	0.93	<0.01^*^
AAMA	0.03 (−0.02, 0.09)	0.25	0.05 (−0.05, 0.14)	0.34	0.59	0.14 (−0.04, 0.32)	0.12	0.03 (−0.02, 0.08)	0.30	<0.01^*^
AMCC	0.03 (−0.02, 0.09)	0.25	0.05 (−0.05, 0.14)	0.34	0.59	0.14 (−0.04, 0.32)	0.12	0.03 (−0.02, 0.08)	0.30	<0.01^*^
ATCA	−0.01 (−0.07, 0.04)	0.60	0.03 (−0.06, 0.11)	0.53	0.76	−0.02 (−0.16, 0.11)	0.74	0.00 (−0.05, 0.05)	0.97	0.79
SBMA	−0.03 (−0.08, 0.01)	0.16	0.05 (−0.03, 0.12)	0.23	0.11	0.08 (−0.06, 0.21)	0.26	−0.02 (−0.07, 0.02)	0.26	0.06
BPMA	−0.02 (−0.06, 0.01)	0.24	−0.01 (−0.07, 0.05)	0.77	0.98	−0.13 (−0.24, −0.03)	0.01^*^	0.00 (−0.03, 0.03)	0.87	<0.01^*^
CEMA	0.05 (0.00, 0.11)	0.07	0.17 (0.07, 0.26)	<0.01^*^	0.24	0.09 (−0.08, 0.26)	0.29	0.06 (0.01, 0.11)	0.01^*^	0.33
CYMA	−0.02 (−0.06, 0.02)	0.35	0.07 (0.01, 0.14)	0.03^*^	0.18	0.10 (−0.03, 0.23)	0.11	0.01 (−0.02, 0.05)	0.43	0.03^*^
DHBMA	−0.06 (−0.13, 0.01)	0.07	0.10 (−0.02, 0.22)	0.09	0.04^*^	0.08 (−0.14, 0.30)	0.49	−0.04 (−0.10, 0.02)	0.22	0.11
2HPMA	−0.07 (−0.11, −0.02)	0.01^*^	0.04 (−0.04, 0.12)	0.36	0.13	−0.09 (−0.23, 0.06)	0.23	−0.02 (−0.07, 0.02)	0.25	0.87
3HPMA	0.01 (−0.05, 0.06)	0.83	0.13 (0.04, 0.22)	<0.01^*^	0.09	0.10 (−0.07, 0.27)	0.24	0.04 (−0.01, 0.09)	0.11	0.11
MA	−0.06 (−0.12, 0.00)	0.06	0.08 (−0.03, 0.19)	0.16	0.03^*^	0.19 (0.00, 0.38)	0.05^*^	−0.05 (−0.10, 0.01)	0.10	<0.01^*^
MHBMA3	0.00 (−0.05, 0.06)	0.98	0.18 (0.09, 0.27)	<0.01^*^	0.03^*^	0.23 (0.06, 0.39)	0.01^*^	0.04 (−0.01, 0.09)	0.09	<0.01^*^
PGA	−0.07 (−0.14, −0.01)	0.02^*^	0.04 (−0.08, 0.15)	0.55	0.24	0.13 (−0.08, 0.34)	0.23	−0.06 (−0.12, 0.00)	0.05	0.01^*^
HMPMA	−0.01 (−0.07, 0.05)	0.75	0.15 (0.05, 0.25)	<0.01^*^	0.04^*^	0.24 (0.07, 0.41)	0.01^*^	0.02 (−0.04, 0.07)	0.50	<0.01^*^

The model was adjusted for age, gender, race, education, family income ratio, serum cotinine, alcohol, smoke, hypertension, diabetes, TC and LDL. *means significant results.

**Figure 5:**
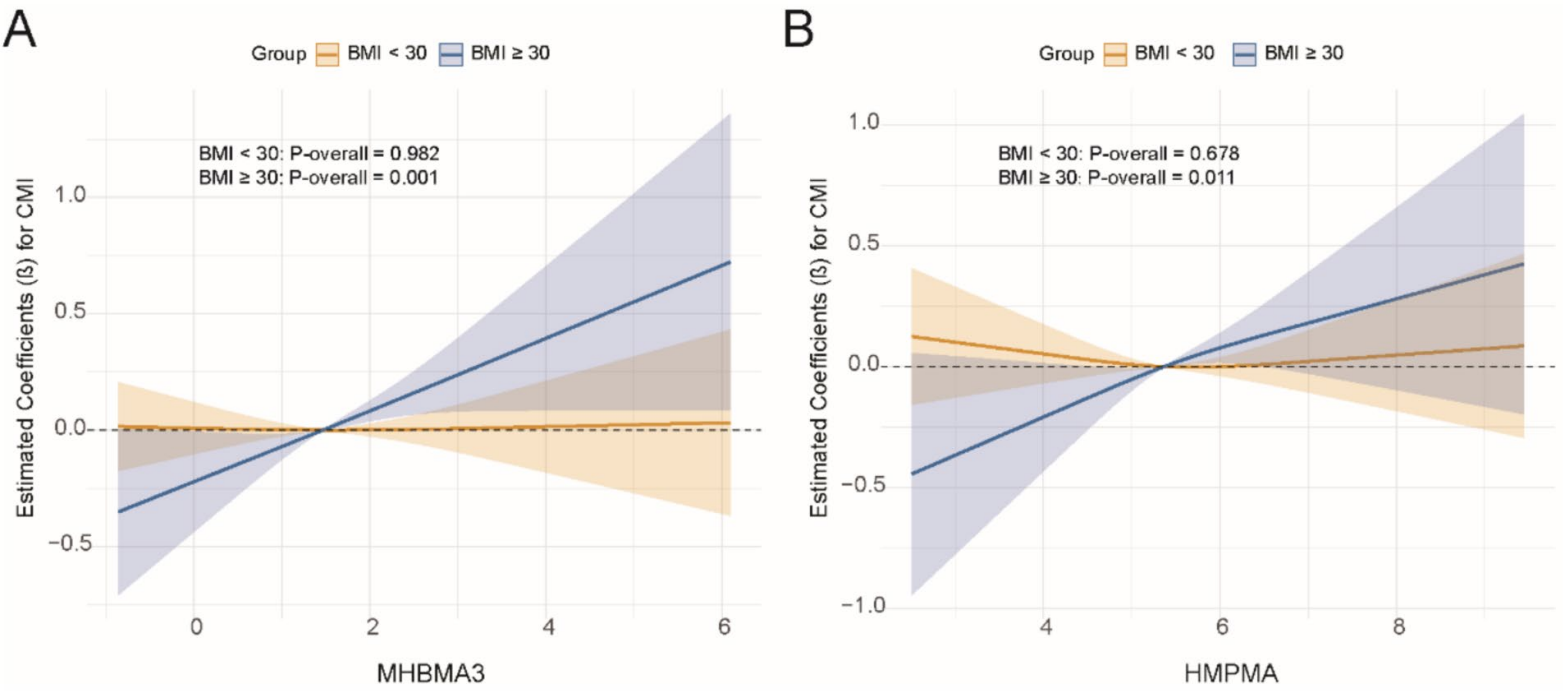
The dose–response relationship curve stratified by BMI using RCS. **(A)** MHBMA3; **(B)** HMPMA.

**Figure 6:**
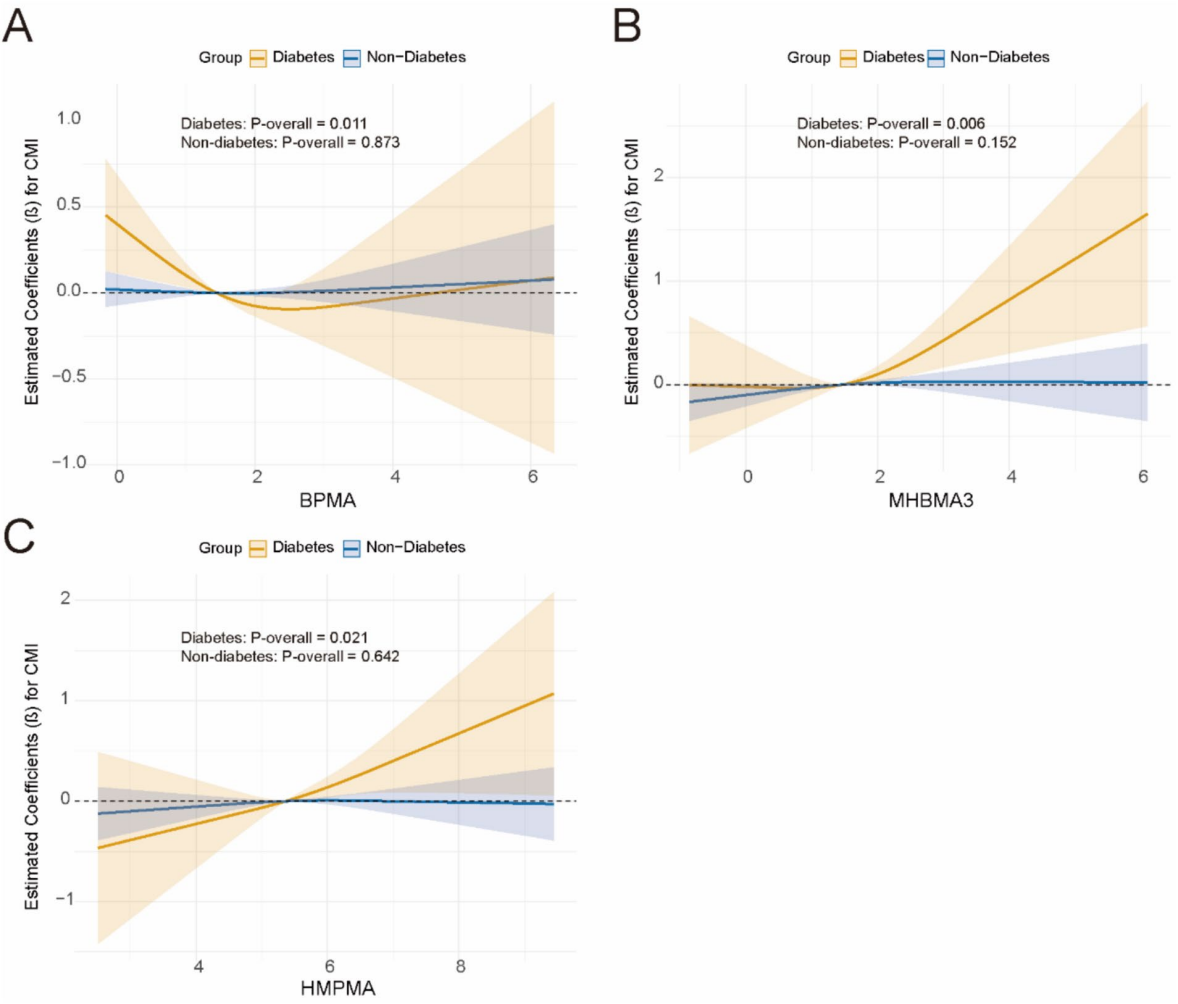
The dose–response relationship curve stratified by diabetes using RCS. **(A)** BMMA; **(B)** MHBMA3; **(C)** HMPMA.

Subsequently, for VOCs that demonstrated interaction effects in the stratified analysis, we applied restricted cubic splines (RCS) to model the dose–response relationships between VOCs and CMI stratified by BMI and diabetes status. Significant results were visualized in Figures 5, 6. The results showed a positive dose–response relationship between MHBMA3 and HMPMA with CMI in individuals with BMI ≥ 30 (*p* = 0.001; *p* = 0.011, respectively). Similarly, among individuals with diabetes, MHBMA3 and HMPMA exhibited positive dose–response relationships with CMI (*p* = 0.006; *p* = 0.021, respectively), while BPMA demonstrated a negative dose–response relationship with CMI (*p* = 0.011). These findings are generally consistent with the earlier results.

### 3.7 Multiple comparison

After rigorously controlling for the risk of false positives due to multiple comparisons using the Benjamini-Hochberg FDR correction, the results are presented in Supplementary Figure S6. Notably, CEMA (*β* = 0.141, 95% CI: 0.088–0.195, FDR < 0.001) and MHBMA3 (*β* = 0.096, 95% CI: 0.044–0.149, FDR = 0.002) were significantly associated with elevated CMI. In addition, 3HPMA (FDR = 0.016), AMCC (FDR = 0.026), and HMPMA (FDR = 0.026) exhibited suggestive associations. All other detected VOCs did not reach the threshold for statistical significance (FDR > 0.05).

## 4 Discussion

This study aimed to explore the association between VOCs and the CMI using data from 2,619 participants. The findings from this research provide significant new insights into the role that VOCs might play in influencing cardiovascular and metabolic health. Our results suggest a clear association between certain VOCs and CMI. Specifically, the higher concentrations of CEMA, 3HPMA, MHBMA3, MPAH, and PFNA in urine were significantly positively associated with CMI in fully adjusted models. This supports the hypothesis that exposure to specific VOCs could potentially influence cardiovascular metabolic dysfunction, which is often reflected in the CMI (24). In addition, a linear relationship was observed between AMCC, CEMA, 3HPMA, MHBMA3, HMPMA, and CMI, further confirming that VOCs contribute to altered metabolic outcomes.

With the ongoing urbanization process, VOC concentrations in work and living environments continue to rise. These chemicals enter the human body through the air, posing serious health risks, particularly to the cardiovascular system (25, 26). Recent studies have shown that long-term exposure to high concentrations of VOCs is closely associated with the development of various cardiovascular diseases. A national study in the United States found that urinary VOC levels were significantly related to CVD (27). In another prospective cohort, individual and combined exposure to VOCs was found to increase the risk of CVD mortality, while reducing exposure to VOCs decreased overall and cardiovascular disease-related mortality (28). In a cross-sectional study involving 49,504 participants, VOCs were confirmed to be significantly associated with CVD after full adjustment (22). Furthermore, a case–control study provided evidence linking exposure to certain household and commercial VOCs with coronary artery disease. A study from China found that increased concentrations of specific VOCs were significantly associated with higher emergency hospital admission rates for cardiovascular diseases (29). In this study, the observed effect size between CEMA, 3HPMA, and CMI suggests that VOC exposure may substantially elevate CMI levels, potentially pushing individuals beyond established high-risk thresholds. Given that higher CMI values have been associated with increased risk of metabolic syndrome and cardiovascular disease, this association highlights the clinical relevance of VOC exposure in contributing to adverse cardiometabolic outcomes. Even at lower levels of VOCs in the environment, cardiovascular damage was still evident. Most of the current research focuses on the direct relationship between VOC exposure and cardiovascular diseases; however, the potential effects on cardiovascular metabolic function remain unclear. This study provides evidence on the association between VOC exposure and CMI, which is one of the highlights of this research. Further research is warranted to explore these findings in longitudinal cohorts to assess long-term risk trajectories.

This study identified a nonlinear relationship between certain VOC exposures and CMI, with AMCC and SBMA exhibiting J-shaped and inverted U-shaped associations, respectively. At low concentrations, AMCC is likely metabolized into less toxic products via detoxification pathways mediated by hepatic CYP450 enzymes, resulting in minimal impact on cardiometabolic function, reflected by the relatively flat curve on the left side of the inflection point (8). However, when exposure exceeds the metabolic capacity, unmetabolized AMCC may induce ROS production through NADPH oxidase, thereby disrupting energy metabolism, leading to a sharp increase on the right side of the curve (27, 30, 31). Moreover, although this study observed an inverted U-shaped association between SBMA and cardiometabolic function, no established mechanisms currently explain the effects of SBMA. Our findings underscore the need for further research in this area. Although some nonlinear patterns are biologically reasonable, unmeasured confounding factors or exposure misclassification may affect the observed curves (32). Future research should incorporate longitudinal exposure assessments and multi-omics data to validate these thresholds and further elucidate the underlying mechanisms. These studies can partly explain the interactive relationship between obesity or diabetes and VOC and CMI.

Based on the subgroup analysis results, we suggest that VOCs may influence metabolic risk factors such as obesity and diabetes, directly or indirectly, further impacting the CMI. Studies have shown that exposure to specific VOCs is positively correlated with an increased BMI (33). Elevated BMI may affect heart metabolic function by increasing blood glucose, lipid levels (such as TG and LDL), and insulin resistance (34–36). Therefore, obesity could amplify the negative effects of VOCs on metabolic health, making VOC exposure’s impact on CMI more pronounced. Adipose tissue in obese individuals continuously releases pro-inflammatory cytokines, which may enhance VOC toxicity through increased oxidative stress (37). Inflammatory signaling upregulates, which, in combination with ROS generated from VOC metabolism, causes vascular endothelial damage. Inflammatory factors downregulate hepatic CYP450 enzyme activity, slowing VOC metabolism and prolonging systemic exposure (38, 39). Furthermore, exposure to VOCs can induce insulin resistance and glucose homeostasis disruption, leading to the onset of diabetes (40). The effects of VOCs on fat metabolism may also be closely related to the development of diabetes. Studies have shown that the accumulation of adipocytes and liver fat, along with increased visceral fat, can impair insulin metabolism, leading to uncontrolled blood glucose levels (41). Diabetic individuals, due to insulin resistance, unstable blood glucose, and metabolic abnormalities, often exhibit higher CMI values. VOC exposure may exacerbate this metabolic disorder, affecting the balance between glucose and lipid metabolism. VOC metabolites may directly inhibit tyrosine phosphorylation of insulin receptor substrate 1, worsening IR (42). IR-related mitochondrial dysfunction may impair cellular repair mechanisms, reducing the ability to mitigate VOC-induced oxidative damage (43). VOC exposure experiments in high-fat diet or diabetic mice to measure inflammatory markers and insulin signaling proteins (44). These results suggest that VOCs may exacerbate cardiometabolic risk by promoting metabolic disturbances associated with obesity and diabetes, such as insulin resistance, inflammation, and oxidative stress. Individuals with obesity and diabetes are more vulnerable to the adverse effects of VOCs due to their pre-existing metabolic abnormalities and inflammatory states. However, further research is needed to elucidate the underlying mechanisms.

Disruption of cardiac metabolic function is often closely associated with cardiovascular diseases such as metabolic syndrome, diabetes, hypertension, and atherosclerosis (45). The potential mechanisms by which VOCs lead to abnormal cardiac metabolic function are not yet fully understood. VOCs may affect cardiac metabolic function directly or indirectly by activating oxidative stress. There is evidence suggesting that long-term exposure to certain VOCs leads to increased levels of oxidative stress markers in the body, which in turn damages cardiovascular endothelial cells, promotes lipid oxidation, and contributes to atherosclerosis (46). Evidence shows that longer working hours are associated with higher levels of MDA and glutathione S-transferase (GST) in workers exposed to benzene, indicating an increased risk of oxidative damage (47). *In vitro* studies have also validated the effectiveness of these biomarkers and provided deeper insights into the potential molecular mechanisms underlying VOC-induced toxicity. For example, oxidative stress was activated in normal human cell lines, with ROS levels increasing in a dose-dependent manner and NAD(P)H quinone dehydrogenase 1 (NQO-1) levels decreasing. This demonstrated that exposure to 1,4-benzoquinone (a benzene metabolite) induces autophagy and apoptosis, leading to hematotoxicity (48). Moreover, oxidative stress caused by VOCs may also influence lipid metabolism by inducing LDL oxidation, thus increasing the risk of atherosclerosis (49). VOC exposure can also trigger chronic low-grade inflammation in the body, which is considered one of the underlying factors of CVD and metabolic disorders. Studies have shown that VOCs activate the immune system by stimulating T-cells to release inflammatory mediators, such as tumor necrosis factor-alpha (TNF-*α*) and interleukins-4 (IL-4) and interleukins-13 (IL-13), damaging endothelial cells and impairing vascular function, which increases the risk of arteriosclerosis (50). Exposure to acrolein induces increased secretion of IL-6 and TNF-α in human vascular endothelial cells (HUVECs), mediated through the NF-κB pathway (51). Animal experiments have shown that VOC exposure increases CD4 + and CD8 + T cell populations in mice and induces gene expression changes associated with apoptosis, oxidative stress, and inflammatory cytokine production (52, 53). VOCs may also cause chronic inflammation in liver tissue, leading to lipid metabolism disorders (54). Abnormal lipid metabolism may be a key factor in impaired cardiac metabolic function. Some studies have used breath analysis to map the VOC profile in human adipocytes, suggesting that adipose tissue enzymes may play an important role in metabolic regulation (55). Certain VOC exposures may lead to the accumulation of fat tissue in the body, especially an increase in visceral fat, which is associated with abdominal obesity (56). While existing studies suggest a potential link between VOCs and disruption of cardiac metabolic function, mechanistic research in this area remains limited, and larger-scale population studies combined with more in-depth *in vivo* and *in vitro* experimental research are needed.

Our findings highlight the need for effective public health strategies to mitigate the impact of VOC exposure on cardiometabolic health. For source control, mandatory VOC emission standards should be established for building materials and furniture, with the promotion of low-emission alternatives. In high-risk areas, enhanced ventilation and real-time air quality monitoring should be implemented to reduce indoor VOC levels. For metabolically sensitive populations (BMI ≥ 30 and diabetes), urinary VOC metabolites should be included as optional tests during annual check-ups to facilitate early detection and timely intervention. For public education and protection of vulnerable groups, awareness of VOC-related risks should be raised, emphasizing proper ventilation and the use of low-VOC products. Regular screening of vulnerable populations, such as pregnant women, the older adult, and children, should be conducted to enable early detection of exposure. Additionally, a national VOC monitoring system should be established to track exposure trends and assess the effectiveness of implemented policies, ensuring timely improvements.

This study has several innovative aspects. First, while many existing studies focus on the relationship between common metabolic risk factors and the CMI, this study innovatively centers on the relationship between VOC exposure and CMI. Second, most existing studies directly examine the relationship between VOCs and CVD, while our research incorporates data on blood lipid status, HDL-C, TG, and waist-to-height ratio, using CMI as a comprehensive health indicator. This allows for a more precise assessment of the potential impact of VOC exposure on metabolic function, providing a more detailed analysis of the effects of environmental pollution on cardiac metabolic health. Finally, this study utilized various statistical models and data analysis methods to conduct a comprehensive and systematic analysis of the influence of individual and mixed VOCs on CMI, thereby expanding the research area on the impact of VOCs on cardiac metabolic health.

However, this study also has some limitations. This study is based on cross-sectional data from NHANES, which limits the ability to establish causality between VOC exposure and cardiometabolic function. Temporal ambiguity prevents us from determining whether VOC exposure precedes changes in cardiometabolic health or vice versa. Additionally, the possibility of reverse causality cannot be excluded, where pre-existing cardiometabolic dysfunction may influence VOC metabolism or retention. To further verify our findings, prospective cohort studies or clinical trials are needed. Second, participants may not have accurately recalled or reported their VOC exposure history or other health conditions, leading to recall bias. Third, the study can only provide information on the relationship between chemicals and health status at a single time point, and it cannot assess the cumulative effects of long-term exposure on health. Fourth, while several known confounders can be adjusted for, it is not possible to fully adjust for all potential confounders. Fourth, using a single spot urine sample may not fully capture long-term VOC exposure, potentially leading to exposure misclassification. To mitigate this, future studies should consider repeated measurement to improve exposure assessment accuracy. Furthermore, although our statistical models accounted for mixture effects, we acknowledge that potential synergistic or antagonistic biological interactions among VOCs were not comprehensively explored. We emphasized the need for future mechanistic studies to investigate these complex interactions.

Our findings underscore the importance of understanding the role of environmental exposures, such as VOCs, in influencing cardiovascular and metabolic health. The results of this study can inform public health policies aimed at reducing VOC exposure, particularly in urban and occupational settings where VOC concentrations tend to be higher. Further research into the mechanisms by which VOCs affect metabolic health will help to identify more effective interventions to mitigate the harmful effects of these pollutants.

In conclusion, this study adds to the growing body of evidence linking VOC exposure to metabolic dysfunction. By highlighting the significant relationship between VOCs and CMI, particularly in individuals with higher BMI or diabetes, we provide crucial information that can inform future research, public health strategies, and policy interventions aimed at reducing VOC-related health risks.

## 5 Conclusion

This study reveals a significant association between specific VOCs and the CMI. We found that known risk factors, such as obesity and diabetes, may play an important role in the relationship between these chemicals and CMI. This finding provides new epidemiological evidence to help us understand the potential mechanisms through which VOCs influence cardiovascular and metabolic function. More importantly, these results lay the foundation for further toxicological and mechanistic studies.

## Data Availability

Publicly available datasets were analyzed in this study. This data can be found here: https://www.cdc.gov/nchs/nhanes/index.html.
