# Supplementary material for: Association between volatile organic compounds exposure and cardiometabolic function: a population-based study
Source: Front Public Health. 2025 Apr 23;13:1570752. doi: 10.3389/fpubh.2025.1570752 (PMC12055860; doi:10.3389/fpubh.2025.1570752)
Supplement: Supplementary file 1 [file Data_Sheet_1.pdf]

Supplementary material

Association between volatile organic compounds exposure and cardiometabolic function: a population-based study

Qiuyu Wang [1], Yongping Cao [2], Fei Ma [2], Hengyang Zhang [2], Yuelin Hu [1 *], Wenwen Xiao [2 *]

1. Department of Electrocardiology, The Second Affiliated Hospital of Wannan Medical College, Wuhu 241000, Anhui, China.

2. Eastern Theater Command Centers for Disease Control and Prevention, Nanjing, China.

[*]**Corresponding Author:** Wenwen Xiao, Eastern Theater Command Centers for Disease Control and Prevention, 293 Zhongshan East Rd, Nanjing, China; Yuelin Hu, Department of Electrocardiology, The Second Affiliated Hospital of Wannan Medical College, Wuhu 241000, Anhui, China.

E-mail addresses: wenwenxiao1996@163.com; hyl04220429@163.com.

Contents

Table S1. Full names and abbreviations of VOCs

Table S2. Distribution of the selected VOCs.

Table S3. Multiple linear regression between individual VOCs and CMI.

Table S4. WQS model regression between mixed VOCs and CMI.

Table S5. Subgroup analysis for association between VOCs and CMI.

Fig. S1. Spearman correlation of VOCs.

Fig. S2. BKMR analysis on VOCs exposure and CMI.

Fig. S3. The univariate exposure-response function (95% CI) between the concentration of selected chemicals and CMI.

Fig. S4. Combined effect of VOCs on CMI in Q-gcomp regression.

Fig. S5. Multiple comparison results using FDR.

Table S1. Full names and abbreviations of VOCs

Variables	Full name
2MHA	2-methylhippuric acid
3-4MHA	3- and 4-methylhippuric acid
AAMA	N-acetyl-S-(2-carbamoylethyl)-l-cysteine
AMCC	N-acetyl-S-(N-methylcarbamoyl)-l-cysteine
ATCA	2-aminothiazoline-4-carboxylic acid
SBMA	N-acetyl-S-(benzyl)-l-cysteine
BPMA	N-acetyl-S-(n-propyl)-l-cysteine
CEMA	N-acetyl-S-(2-carboxyethyl)-l-cysteine
CYMA	N-acetyl-S-(2-cyanoethyl)-l-cysteine
DHBMA	N-acetyl-S-(3,4-dihydroxybutyl)-l-cysteine
2HPMA	N-acetyl-S-(2-hydroxypropyl)-l-cysteine
3HPMA	N-acetyl-S-(3-hydroxypropyl)-l-cysteine
MA	mandelic acid
MHBMA3	N-acetyl-S-(4-hydroxy-2-butenyl)-l-cysteine
PGA	phenylglyoxylic acid
HMPMA	N-acetyl-S-(3-hydroxypropyl-1-methyl)-l-cysteine

Table S2. Distribution of the selected VOCs.

Variables	Min	P5	P10	P25	P50	P75	P90	P95	Max	LOD
2MHA	3.54	3.54	5.6	12.55	30.6	78.55	162.2	222	1660	91.5
3-4MHA	5.66	28.79	41.64	85.9	204	562	1140	1600	20400	99.7
AAMA	1.56	10.8	16.4	28.5	53.9	110	204	297.1	2750	99.9
AMCC	4.43	28.4	42.78	82.8	158	318	588	836.2	46300	99.9
ATCA	10.6	20.9	23.68	52.2	108	208.5	373.6	515	2090	93.1
SBMA	0.35	1.51	2.15	3.68	6.75	13.35	25.94	45.11	1040	99.8
BPMA	0.85	0.85	0.85	1.51	4.22	11.6	29.62	49.7	566	78.8
CEMA	4.92	18.8	28.2	53.7	101	186	338	451.1	2240	99.4
CYMA	0.35	0.35	0.35	0.86	1.7	14.35	151	259.1	1620	87.3
DHBMA	17.1	82.78	114	198	329	508	721.4	877.1	3890	100
2HPMA	3.75	6.68	9.90	17.4	32.6	64	120	194.2	9360	97
3HPMA	9.2	47.75	70.28	125	229	451	1030	1661	14600	99.9
MA	8.5	33.98	45.78	79.6	136	227.5	371	493.1	5170	99.2
MHBMA3	0.42	0.84	1.28	2.36	4.33	9.12	28.8	51.55	444	97.4
PGA	8.5	55.59	78.18	136	228	367	552.6	725.1	2080	99.9
HMPMA	12.3	51.69	72.28	125	214	390	989.4	1791	12600	100

LOD, limit of detection.

Table S3. Multiple linear regression between individual VOCs and CMI.

	Model1		Model2	
Variable	Estimate (95% CI)	P value	Estimate (95% CI)	P value
2MHA	0.01 (-0.03, 0.05)	0.76	0.02 (-0.02, 0.06)	0.36
3-4MHA	0.01 (-0.03, 0.05)	0.72	0.01 (-0.03, 0.05)	0.57
AAMA	0.01 (-0.04, 0.06)	0.63	0.02 (-0.03, 0.08)	0.36
AMCC	0.08 (0.04, 0.13)	<0.01*	0.07 (0.02, 0.13)	0.01*
ATCA	0.04 (-0.01, 0.09)	0.15	0.02 (-0.02, 0.07)	0.30
SBMA	0.02(-0.03, 0.07)	0.42	0.03 (-0.02, 0.07)	0.28
BPMA	-0.01 (-0.05, 0.02)	0.47	0.00 (-0.04, 0.03)	0.88
CEMA	0.14 (0.08, 0.19)	<0.01*	0.15 (0.09, 0.20)	<0.01*
CYMA	0.02 (-0.01, 0.04)	0.19	0.02 (-0.02, 0.06)	0.38
DHBMA	0.06 (-0.01, 0.13)	0.11	0.05 (-0.01, 0.12)	0.11
2HPMA	-0.01 (-0.06, 0.03)	0.56	-0.01 (-0.06, 0.04)	0.72
3HPMA	0.07 (0.03, 0.12)	<0.01*	0.08 (0.03, 0.14)	<0.01*
MA	0.05 (-0.01, 0.11)	0.11	0.03 (-0.04, 0.09)	0.40
MHBMA3	0.07 (0.03, 0.12)	<0.01*	0.10 (0.05, 0.15)	<0.01*
PGA	0.01 (-0.06, 0.07)	0.87	0.00 (-0.07, 0.06)	0.97
HMPMA	0.09 (0.04, 0.14)	<0.01*	0.08 (0.02, 0.14)	0.01*

CI, confidence interval. Model1 adjusted for age, gender, race, educational levels, and family income ratio. Model2 further adjusted for alcohol, smoking, hypertension, diabetes, BMI, serum cotinine, LDL, and total cholesterol.

Table S4. WQS model regression between mixed VOCs and CMI.

Exposures	β (95% CI)	P value
Positive	0.02 (-0.04, 0.09)	0.47
Negative	-0.01 (-0.09, 0.06)	0.71

CI, confidence interval. The model was adjusted for age, gender, race, educational levels, and family income ratio, alcohol, smoking, hypertension, diabetes, BMI, serum cotinine, LDL, and total cholesterol.

Table S5. Subgroup analysis for association between VOCs and CMI.

Age	<55		>55		
	Estimate (95% CI)	P value	Estimate (95% CI)	P value	P-int
2MHA	0.02 (-0.02, 0.07)	0.31	0.01 (-0.05, 0.08)	0.64	0.78
3-4MHA	0.03 (-0.02, 0.07)	0.23	0.01 (-0.05, 0.07)	0.80	0.91
AAMA	0.02 (-0.04, 0.08)	0.46	0.07 (-0.01, 0.15)	0.11	0.14
AMCC	0.02 (-0.04, 0.08)	0.46	0.07 (-0.01, 0.15)	0.11	0.14
ATCA	-0.02 (-0.08, 0.03)	0.43	0.03 (-0.05, 0.10)	0.48	0.29
SBMA	-0.03 (-0.08, 0.02)	0.18	0.02 (-0.04, 0.09)	0.49	0.16
BPMA	0.00 (-0.04, 0.04)	0.86	-0.03 (-0.08, 0.03)	0.32	0.55
CEMA	0.06 (0.01, 0.12)	0.03*	0.08 (0.00, 0.17)	0.04*	0.41
CYMA	0.01 (-0.03, 0.05)	0.64	0.04 (-0.02, 0.10)	0.22	0.09
DHBMA	-0.01 (-0.08, 0.06)	0.75	-0.03 (-0.14, 0.07)	0.53	0.98
2HPMA	-0.02 (-0.07, 0.03)	0.39	-0.04 (-0.10, 0.03)	0.31	0.81
3HPMA	0.05 (-0.01, 0.11)	0.08	0.05 (-0.04, 0.13)	0.28	0.40
MA	-0.02 (-0.09, 0.04)	0.50	-0.01 (-0.10, 0.09)	0.89	0.49
MHBMA3	0.05 (0.00, 0.11)	0.06	0.09 (0.01, 0.18)	0.04*	0.14
PGA	-0.02 (-0.09, 0.05)	0.54	-0.05 (-0.15, 0.05)	0.30	0.94
HMPMA	0.05 (-0.01, 0.11)	0.13	0.05 (-0.05, 0.14)	0.33	0.44
Gender	Male		Female		
	Estimate (95% CI)	P value	Estimate (95% CI)	P value	P-int
2MHA	0.03 (-0.03, 0.09)	0.29	0.01 (-0.03, 0.06)	0.52	0.56
3-4MHA	0.03 (-0.03, 0.09)	0.35	0.01 (-0.03, 0.05)	0.55	0.70
AAMA	0.05 (-0.03, 0.13)	0.23	0.04 (-0.01, 0.09)	0.15	0.94
AMCC	0.05 (-0.03, 0.13)	0.23	0.04 (-0.01, 0.09)	0.15	0.94
ATCA	-0.02 (-0.08, 0.05)	0.61	-0.01 (-0.06, 0.05)	0.83	0.56
SBMA	-0.04 (-0.10, 0.03)	0.27	0.01 (-0.04, 0.05)	0.76	0.99
BPMA	-0.03 (-0.08, 0.02)	0.25	0.00 (-0.04, 0.03)	0.86	0.19
CEMA	0.09 (0.01, 0.17)	0.02*	0.06 (0.01, 0.12)	0.02*	0.80
CYMA	0.00 (-0.05, 0.05)	0.97	0.04 (0.00, 0.09)	0.06	0.03*
DHBMA	-0.04 (-0.13, 0.06)	0.48	-0.01 (-0.08, 0.06)	0.82	0.96
2HPMA	-0.02 (-0.09, 0.05)	0.52	-0.02 (-0.07, 0.02)	0.32	0.37
3HPMA	0.05 (-0.03, 0.13)	0.23	0.06 (0.00, 0.11)	0.05*	0.27
MA	-0.06 (-0.14, 0.03)	0.17	0.01 (-0.05, 0.08)	0.65	0.20
MHBMA3	0.08 (0.00, 0.15)	0.05*	0.06 (0.00, 0.11)	0.05*	0.42
PGA	-0.05 (-0.14, 0.04)	0.24	-0.01 (-0.08, 0.05)	0.70	0.27
HMPMA	0.04 (-0.04, 0.12)	0.34	0.06 (0.00, 0.12)	0.06	0.35
Race	Non-Hispanic White		Other		
	Estimate (95% CI)	P value	Estimate (95% CI)	P value	P-int
2MHA	0.05 (0.00, 0.11)	0.07	-0.02 (-0.07, 0.03)	0.44	0.01*
3-4MHA	0.06 (0.00, 0.11)	0.05*	-0.03 (-0.08, 0.02)	0.27	<0.01*
AAMA	0.10 (0.03, 0.17)	0.01*	0.01 (-0.05, 0.08)	0.66	<0.01*
AMCC	0.10 (0.03, 0.17)	0.01*	0.01 (-0.05, 0.08)	0.66	<0.01*

ATCA	0.00 (-0.07, 0.06)	0.93	-0.01 (-0.07, 0.04)	0.63	0.54
SBMA	0.01 (-0.06, 0.07)	0.87	-0.06 (-0.11, 0.00)	0.03*	0.15
BPMA	-0.03 (-0.08, 0.02)	0.29	0.00 (-0.04, 0.04)	1.00	0.35
CEMA	0.10 (0.03, 0.17)	0.01*	0.00 (-0.07, 0.06)	0.94	<0.01*
CYMA	0.06 (0.02, 0.11)	0.01*	-0.04 (-0.10, 0.01)	0.09	<0.01*
DHBMA	0.00 (-0.09, 0.10)	0.92	-0.08 (-0.16, 0.00)	0.05	0.01*
2HPMA	-0.01 (-0.07, 0.06)	0.77	-0.05 (-0.11, 0.00)	0.06	0.08
3HPMA	0.09 (0.02, 0.17)	0.01*	0.00 (-0.06, 0.07)	0.93	<0.01*
MA	0.01 (-0.07, 0.10)	0.78	-0.07 (-0.14, 0.00)	0.06	0.01*
MHBMA3	0.11 (0.04, 0.18)	<0.01*	0.00 (-0.06, 0.07)	0.94	<0.01*
PGA	0.03 (-0.06, 0.12)	0.50	-0.11 (-0.18, -0.03)	0.01*	<0.01*
HMPMA	0.09 (0.01, 0.16)	0.03*	-0.01 (-0.08, 0.06)	0.82	<0.01*
Education	Below high school		Other		
	Estimate (95% CI)	P value	Estimate (95% CI)	P value	P-int
2MHA	0.03 (-0.03, 0.09)	0.35	0.01 (-0.03, 0.06)	0.59	0.94
3-4MHA	0.03 (-0.03, 0.09)	0.32	0.01 (-0.04, 0.05)	0.73	0.71
AAMA	0.06 (-0.02, 0.14)	0.17	0.03 (-0.03, 0.10)	0.27	0.67
AMCC	0.06 (-0.02, 0.14)	0.17	0.03 (-0.03, 0.10)	0.27	0.67
ATCA	0.01 (-0.06, 0.07)	0.86	-0.01 (-0.06, 0.05)	0.81	0.67
SBMA	-0.02 (-0.09, 0.04)	0.46	0.00 (-0.06, 0.05)	0.91	0.72
BPMA	-0.01 (-0.06, 0.04)	0.72	-0.02 (-0.06, 0.02)	0.42	0.97
CEMA	0.05 (-0.03, 0.13)	0.19	0.09 (0.03, 0.15)	<0.01*	0.32
CYMA	0.01 (-0.04, 0.07)	0.69	0.04 (-0.01, 0.08)	0.14	0.37
DHBMA	-0.03 (-0.13, 0.07)	0.52	-0.02 (-0.10, 0.06)	0.64	0.71
2HPMA	-0.02 (-0.09, 0.04)	0.49	-0.03 (-0.08, 0.02)	0.23	0.89
3HPMA	0.05 (-0.03, 0.12)	0.24	0.06 (0.00, 0.12)	0.06	0.44
MA	0.00 (-0.09, 0.09)	0.98	-0.03 (-0.10, 0.04)	0.41	0.77
MHBMA3	0.03 (-0.04, 0.11)	0.37	0.10 (0.04, 0.17)	<0.01*	0.13
PGA	-0.07 (-0.16, 0.03)	0.17	-0.01 (-0.09, 0.06)	0.73	0.30
HMPMA	0.03 (-0.05, 0.11)	0.47	0.07 (0.00, 0.13)	0.05*	0.40
Alcohol	Yes		No		
	Estimate (95% CI)	P value	Estimate (95% CI)	P value	P-int
2MHA	0.08 (-0.07, 0.23)	0.30	0.01 (-0.03, 0.05)	0.54	0.91
3-4MHA	0.09 (-0.06, 0.24)	0.24	0.01 (-0.03, 0.04)	0.66	0.76
AAMA	0.01 (-0.18, 0.19)	0.96	0.05 (0.00, 0.10)	0.05*	0.39
AMCC	0.01 (-0.18, 0.19)	0.96	0.05 (0.00, 0.10)	0.05*	0.39
ATCA	-0.06 (-0.22, 0.10)	0.46	0.00 (-0.04, 0.05)	0.85	0.16
SBMA	0.02 (-0.13, 0.16)	0.82	-0.02 (-0.06, 0.02)	0.36	0.17
BPMA	-0.15 (-0.27, -0.03)	0.01*	0.00 (-0.03, 0.03)	0.97	0.01*
CEMA	0.01 (-0.16, 0.18)	0.90	0.08 (0.04, 0.13)	<0.01*	0.26
CYMA	0.06 (-0.05, 0.18)	0.29	0.01 (-0.02, 0.05)	0.51	0.68
DHBMA	-0.16 (-0.37, 0.05)	0.14	0.00 (-0.06, 0.06)	0.97	0.13
2HPMA	-0.13 (-0.29, 0.04)	0.13	-0.01 (-0.05, 0.03)	0.58	0.03*
3HPMA	-0.02 (-0.20, 0.16)	0.83	0.06 (0.01, 0.11)	0.01*	0.18

MA	-0.12 (-0.30, 0.07)	0.22	0.00 (-0.06, 0.05)	0.96	0.07
MHBMA3	0.11 (-0.05, 0.28)	0.18	0.06 (0.01, 0.11)	0.01*	0.79
PGA	-0.18 (-0.39, 0.03)	0.09	-0.01 (-0.07, 0.05)	0.69	0.03*
HMPMA	0.02 (-0.16, 0.20)	0.85	0.05 (0.00, 0.11)	0.05*	0.37
Hypertension	Yes		No		
	Estimate (95% CI)	P value	Estimate (95% CI)	P value	P-int
2MHA	0.02 (-0.06, 0.10)	0.62	0.02 (-0.02, 0.06)	0.40	0.59
3-4MHA	-0.01 (-0.08, 0.07)	0.88	0.02 (-0.02, 0.06)	0.29	0.96
AAMA	0.02 (-0.08, 0.12)	0.70	0.05 (-0.01, 0.10)	0.09	0.76
AMCC	0.02 (-0.08, 0.12)	0.70	0.05 (-0.01, 0.10)	0.09	0.76
ATCA	0.01 (-0.08, 0.09)	0.89	-0.01 (-0.06, 0.04)	0.67	0.61
SBMA	0.01 (-0.07, 0.08)	0.85	-0.02 (-0.07, 0.02)	0.31	0.65
BPMA	-0.02 (-0.09, 0.04)	0.47	-0.01 (-0.04, 0.03)	0.69	0.47
CEMA	0.06 (-0.04, 0.15)	0.26	0.07 (0.02, 0.13)	0.01*	0.59
CYMA	0.01 (-0.06, 0.08)	0.88	0.03 (-0.01, 0.07)	0.22	0.58
DHBMA	-0.06 (-0.19, 0.06)	0.33	-0.01 (-0.07, 0.06)	0.84	0.29
2HPMA	-0.08 (-0.16, 0.01)	0.07	-0.01 (-0.06, 0.04)	0.68	0.22
3HPMA	0.03 (-0.06, 0.13)	0.49	0.05 (0.00, 0.11)	0.06	0.81
MA	-0.02 (-0.13, 0.09)	0.73	-0.02 (-0.08, 0.04)	0.47	0.87
MHBMA3	0.05 (-0.05, 0.15)	0.31	0.07 (0.02, 0.12)	0.01*	0.84
PGA	-0.06 (-0.17, 0.06)	0.32	-0.02 (-0.09, 0.04)	0.47	0.64
HMPMA	0.05 (-0.06, 0.15)	0.39	0.04 (-0.02, 0.10)	0.15	0.52
Smoke	Yes		No		
	Estimate (95% CI)	P value	Estimate (95% CI)	P value	P-int
2MHA	0.05 (-0.02, 0.11)	0.14	-0.01 (-0.05, 0.04)	0.75	0.14
3-4MHA	0.04 (-0.02, 0.11)	0.18	-0.01 (-0.05, 0.04)	0.70	0.16
AAMA	0.08 (0.00, 0.16)	0.05*	0.01 (-0.05, 0.07)	0.68	0.02*
AMCC	0.08 (0.00, 0.16)	0.05*	0.01 (-0.05, 0.07)	0.68	0.02*
ATCA	0.03 (-0.04, 0.09)	0.45	-0.03 (-0.09, 0.03)	0.30	0.20
SBMA	0.00 (-0.07, 0.06)	0.98	-0.02 (-0.07, 0.03)	0.53	0.82
BPMA	-0.04 (-0.09, 0.02)	0.18	0.01 (-0.03, 0.05)	0.75	0.17
CEMA	0.04 (-0.04, 0.12)	0.34	0.09 (0.03, 0.15)	<0.01*	0.63
CYMA	0.04 (-0.01, 0.10)	0.12	-0.01 (-0.06, 0.04)	0.72	0.07
DHBMA	-0.02 (-0.12, 0.08)	0.72	-0.03 (-0.10, 0.05)	0.51	0.52
2HPMA	-0.05 (-0.12 ,0.02)	0.19	-0.02 (-0.07, 0.03)	0.45	0.94
3HPMA	0.03 (-0.04, 0.11)	0.37	0.05 (-0.01, 0.12)	0.10	0.82
MA	0.03 (-0.06, 0.11)	0.57	-0.06 (-0.12, 0.01)	0.10	0.06
MHBMA3	0.10 (0.03, 0.18)	0.01*	0.03 (-0.04, 0.09)	0.39	0.04*
PGA	0.01 (-0.09, 0.10)	0.89	-0.07 (-0.15, 0.00)	0.04*	0.08
HMPMA	0.07 (-0.01, 0.15)	0.07	0.01 (-0.06, 0.08)	0.78	0.05

CI, confidence interval, P-int, p for interaction. The model was adjusted for age, gender, race, educational levels, and family income ratio, alcohol, smoking, hypertension,

diabetes, BMI, serum cotinine, LDL, and total cholesterol.

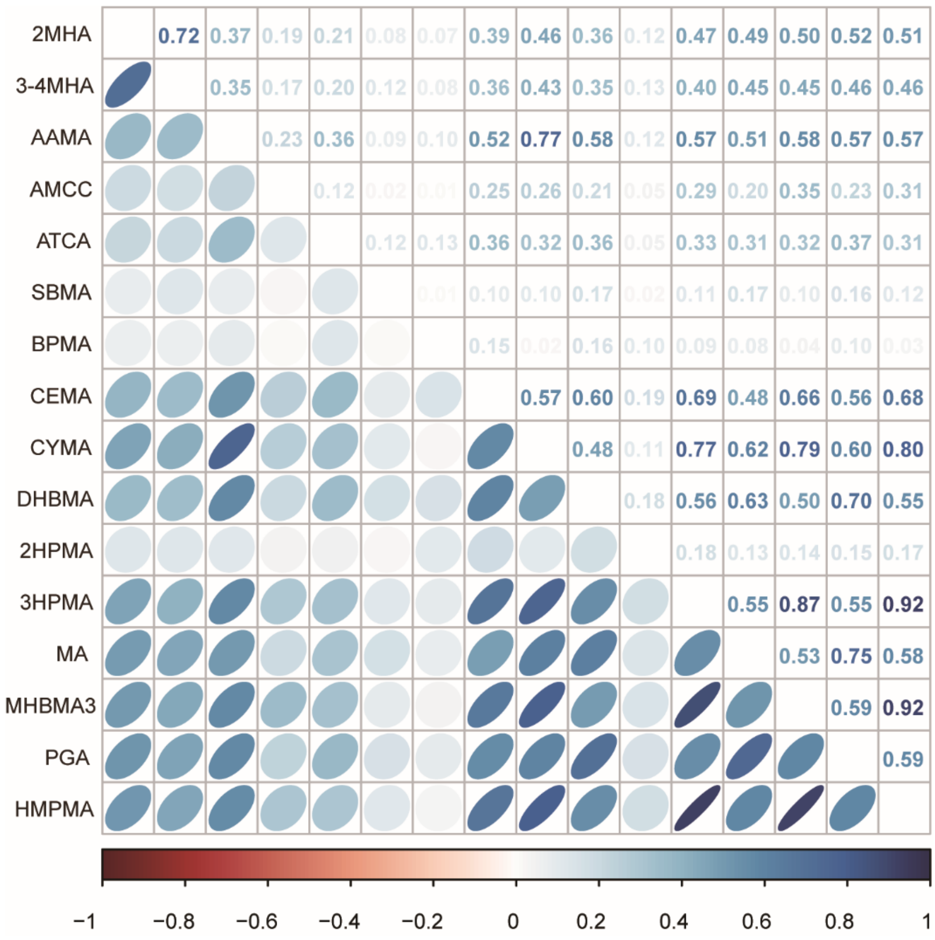

Fig. S1. Spearman correlation of VOCs.

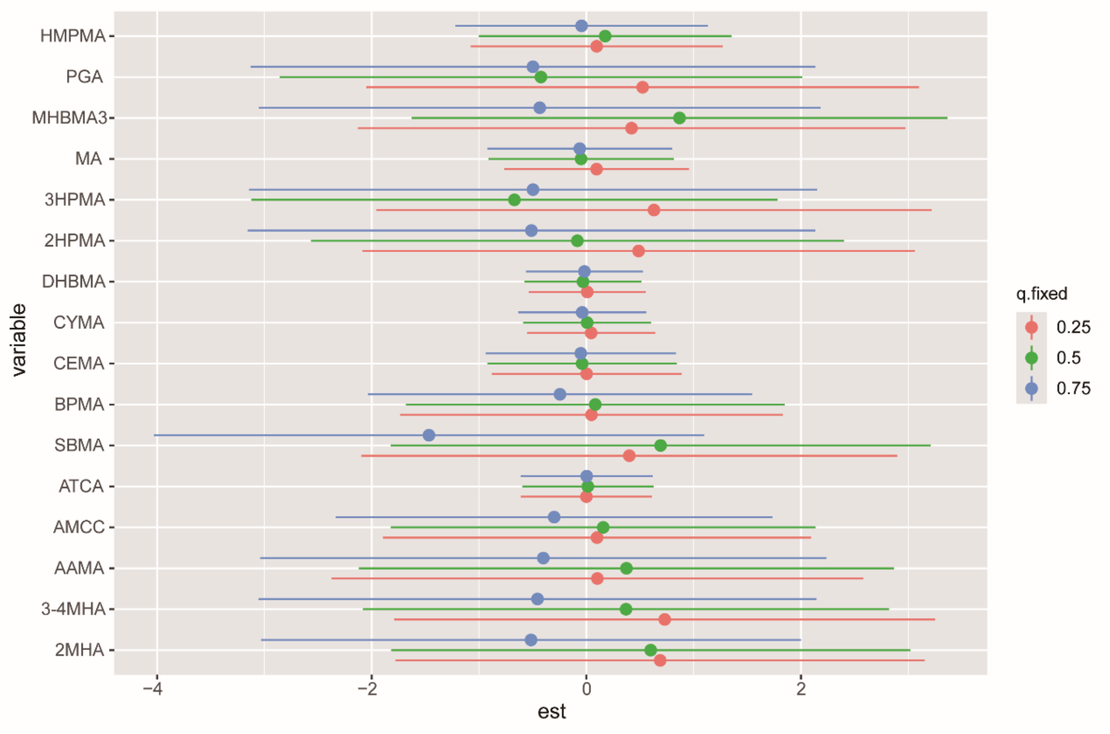

Fig. S2. BKMR analysis on VOCs exposure and CMI. The impact of a single VOC, when the single VOC is in the 75th percentile and the 25th percentile, on the potential continuous outcome of CMI, while all VOCs are in the 25th, 50th, or 75th percentile. "Est" is defined as the association between a single VOC and the potential continuous outcome.

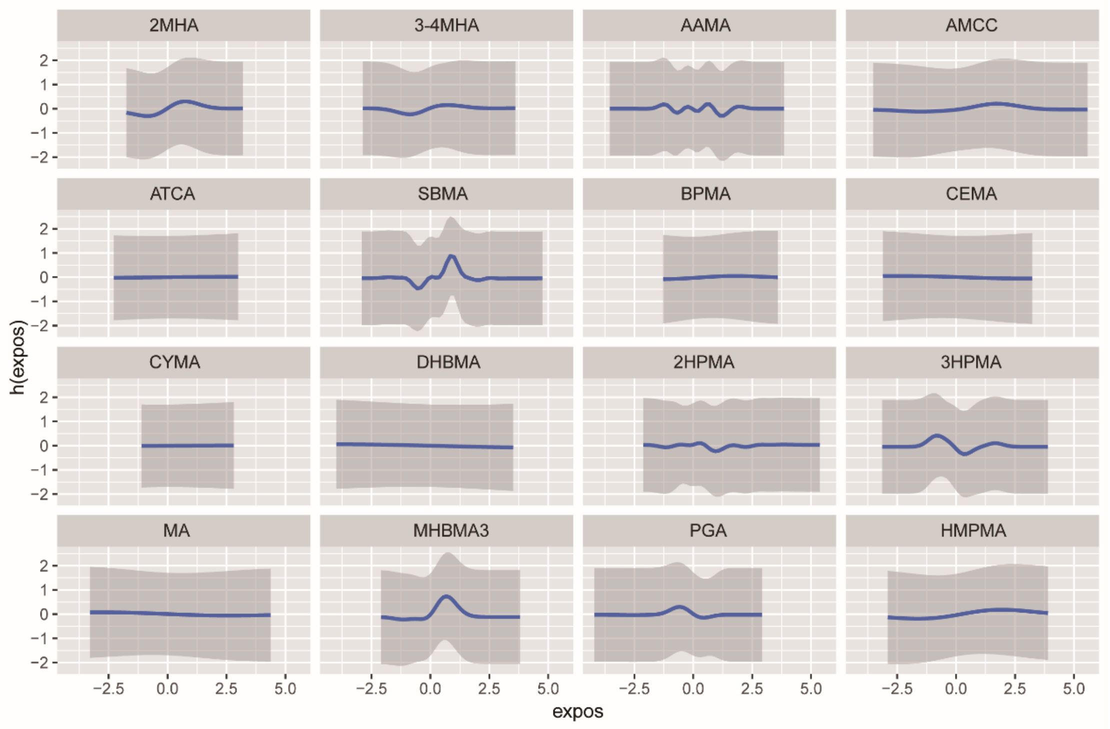

Fig. S3. The univariate exposure-response function (95% CI) between the concentration of selected chemicals and CMI was determined while keeping the concentration of other chemicals at the median.

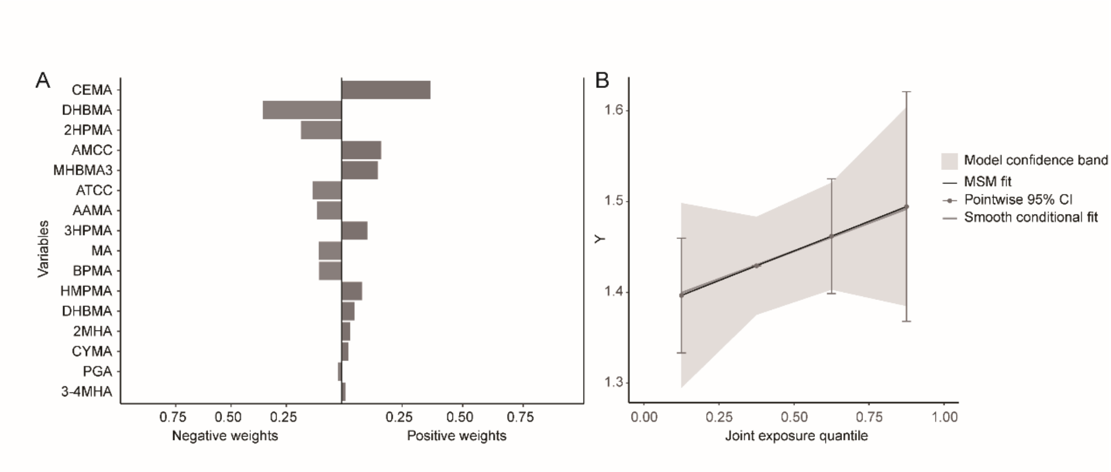

Fig. S4. Combined effect of VOCs on CMI in Q-gcomp regression. (A) The weight of bidirectional mixed effects. (B) The linear relationship between VOC mixed exposure and CMI.

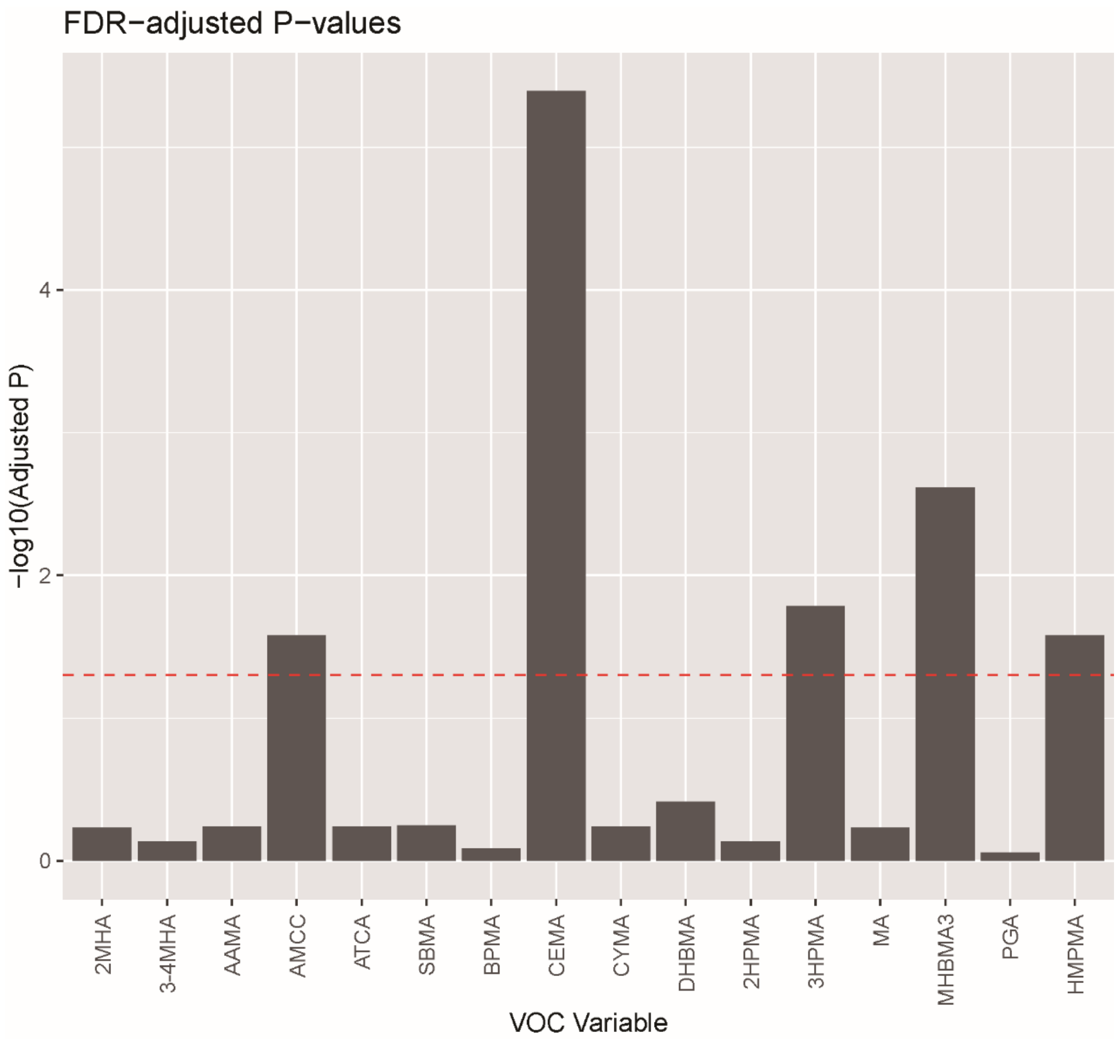

Fig. S5. Multiple comparison results using FDR.
